# Fabrication and Characterization of Highly Porous Gyroid Scaffolds Composed of Deproteinized Bone Mineral

**DOI:** 10.3390/jfb16040119

**Published:** 2025-03-28

**Authors:** Otoniel Durán Hernández, Vail Baumer, Genesis Marrero, Sreya Karumanchi, David Prawel

**Affiliations:** 1 School of Materials Science and Engineering, Colorado State University, Fort Collins, CO 80523, USA; otoniel.duran@colostate.edu; 2 Department of Mechanical Engineering, Colorado State University, Fort Collins, CO 80523, USA; 3 School of Biomedical Engineering, Colorado State University, Fort Collins, CO 80523, USA

**Keywords:** 3D printing, TCP, gyroid, scaffold, robocasting, tissue engineering, deproteinized bone mineral

## Abstract

Current treatment methods for critical bone defects involve the implantation of large bone grafts, which are limited by tissue availability and failure to heal correctly with high complication rates. Bioengineered scaffolds have emerged, which deploy biodegradable, highly osteoconductive materials in porous structures to accommodate the high mass transport requirements of large bone defects. Ideal scaffold biomaterials require a balance between strength, composition, and osteoconduction, a balance which has yet to be discovered. Naturally derived materials like deproteinized bovine bone mineral (DBBM) have seen successful clinical use for decades as bone void fillers, but their granular or putty form lacks the interconnected porosity required to treat large defects. Leveraging the clinical success of DBBM, this paper presents the first fabrication of highly porous scaffolds composed of naturally derived, deproteinized bone mineral, for potential use in large bone defects. Ovine bone mineral powder was prepared from fresh ovine bone, fabricated into a photopolymeric slurry and 3D-printed using a photocasting process into 67% porous gyroid scaffolds. Ovine bone mineral composition, surface microstructure, compressive properties, and failure probability were evaluated and compared to gyroid scaffolds composed of tricalcium phosphate. Both scaffold types were similar, with characteristics in the low range of human cancellous bone.

## 1. Introduction

Bone fractures are one of the most frequent types of hospitalized traumas with reports of about 32.7 million new cases of lower leg fractures globally in 2019 [1,2] accounting for a median of 32 lost days of work per person and an estimated total healthcare cost in excess of USD 81 billion annually [3]. Significant long-bone fractures such as open tibial fractures are often associated with higher complication rates, like non-union or delayed union [4]. These critically sized bone defects are large enough to require surgical intervention because they will never spontaneously heal for a variety of reasons including weight-bearing, disease, adjunct trauma or congenital conditions.

Bone grafts are widely used in the treatment of critical bone defects. The global bone graft and substitute market was valued at USD 2.93 billion in 2024 with a predicted compound annual growth rate of 6.4% [3]. Despite limited success rates, autografts are the gold-standard treatment since they contain the patient’s own osteogenic cells and eliminate immune reaction risks of allografts or xenografts. Unfortunately, autografts are limited by the availability and quantity of harvestable tissue, and they create two surgical sites and cause significant patient discomfort [5]. Allografts are also popular but prone to complications, particularly in large bone defects, including recurring failure to heal correctly [6] and non-union rates as high as 21% [7]. 

Synthetic bone tissue engineering (BTE) scaffolds have emerged in recent decades as promising alternatives to bone grafting, particularly in critical defects. Scaffolds are three-dimensional (3D) structures that have the potential to overcome the limitations of grafting by providing structural support and adequate gas, nutrient, and waste exchange in large bone defects. Complex scaffold topologies like triply periodic minimal surfaces (TPMSs) are providing promising open-cell architectures for bone regeneration [8,9], which are enabling scaffolds that are stiffer and stronger than traditional rectilinear scaffold topologies [10,11,12] of the same material. TPMS topologies such as gyroid [13,14,15] are proving to be ideal candidates for BTE due to their relatively high mechanical energy absorption and robustness [14], continuous, interconnected internal porous structure, high surface area per volume [10,11,13], and improved stiffness to support higher loads. An interconnected porous structure enables higher permeability and perfusion [16,17], which are important factors for cell migration and proliferation. A high surface area per volume is favorable for cell proliferation and growth. Numerous researchers have reported 3D printing of gyroid scaffolds in numerous materials [9,10,13], including some calcium phosphate-based materials like tricalcium phosphate (TCP) [18,19].

Calcium phosphate-based materials are popular for fabricating BTE scaffolds due to their biocompatibility, high levels of osteoconductivity, compositional similarities to human bone mineral, non-immunogenicity, and tunable degradation rates [20]. These materials are bio-absorbable as they release calcium during degradation, which supports bone formation, resulting in excellent osteoconductivity [21]. Numerous trace elements that are found in bone, such as Zn, Mg, Sr, Si and Mn, have been added to TCP scaffolds (also known as “doping”) to improve mechanical properties and bioactivity [22,23,24,25,26] and accelerate new bone formation [27]. Enhanced bioactivity is believed to result from the effect of dopants on local cellular, surface, and signaling processes [23,25,26,28]. Mg plays an important role in bone formation, many aspects of metabolism, and control of many biological functions [29], and Sr has a beneficial effect on bone formation [30]. ZnO doping increases the densification of TCP ceramics [31] and appears to increase growth factors like VEGF and BMP-2 [32,33]. Si promotes biological activity and plays important roles in the proliferation and differentiation of osteoblast-like cells and biomineralization [28,34,35]. Many other minerals may also play a role in bone development, but many have yet to be explored [26]. Unfortunately, considering all the trace elements in bone, the ideal combinations and concentrations of all possible dopants remain elusive and are likely indeterminate due to the large number of naturally occurring minerals in bone at a variety of concentrations. 

Naturally derived materials are popular grafting materials that are widely commercially available for filling bone voids. Deproteinized bone-based materials, also known as anorganic bone mineral (ABM) [36,37] and deproteinized bovine bone mineral (DBBM) [38,39,40], have seen successful clinical use for decades as bone defect void fillers and grafting biomaterials. Unfortunately, their clinical use is limited to small fractures and voids because their particulate or putty form factors lack the interconnected macro-porosity required to treat large defects. High macro-porosity is required to support the influx of mesenchymal cells, osteoblasts, and the development of a vascular supply [41,42,43]. Particulates and pastes also present significant handling, strength, and stability challenges in large bone defects [44]. 

The clinical success of DBBM leads to a hypothesis that highly porous scaffolds composed of pure, naturally derived bone mineral might prove promising as BTE scaffolds because they will already contain the ideal concentrations of the full suite of minerals found in natural bone. The first step in testing this hypothesis, which is presented in this paper, is to fabricate highly porous scaffolds from naturally derived ovine bone mineral (OBM) and evaluate their physical characteristics in the context of BTE. Pure OBM powder was prepared from fresh ovine bone, then a photopolymeric slurry was formulated from this OBM powder and 3D-printed into gyroid scaffolds using a method called photocasting [19,45]. The scaffolds were then sintered into densified gyroid structures and characterized to evaluate their physical characteristics compared to gyroid scaffolds composed of β-TCP.

## 2. Materials and Methods

### 2.1. Powder Processing and Characterization

Compact bone material was harvested from diaphyseal regions of tibia, femur, radius, and metatarsi of healthy sheep at their time of sacrifice from a previous orthopedic biomechanic research project that did not use pharmaceuticals or bioactive agents. All animal-related procedures were approved by the Institutional Animal Care and Use Committee (IACUC No: 2935, approve date 2 December 2021) at Colorado State University. Soft tissues were removed through 24-hour hot water maceration, and then the bones were treated with 16% hydrogen peroxide for 2 hours to remove lipid deposits and debris. The bones were fully air-dried and then band-sawed into half disks with varied thickness (2–4 mm) and radius (4–5 mm). Bones were then cryoground in liquid nitrogen and sintered at 850°C for 2 hours to remove all organic components. The resulting deproteinized ovine bone mineral (OBM) was then wet-milled in 70% ethanol for 10 hours, dried under a 500 W heat lamp until ethanol was fully evaporated, and stored under a vacuum for further processing.

Particle characteristics influence slurry viscosity and scaffold grain size [46]. Therefore, the particle size and shape of β-tricalcium phosphate (TCP) and OBM powders were measured using a field emission electron scanning microscope (SEM) JSM-6500F (JEOL USA, Peabody, MA, USA).

Trace elements in OBM powder were detected through inductively coupled plasma mass spectrometry (ICP-MS). Samples were prepared through microwave digestion of 100 ± 10 mg of OBM powder with 10 mL 0.2% concentrated, redistilled nitric acid. After digestion, samples were successively diluted to approximately 267 ppm before being inserted into the ICP-MS instrument. In addition, sample data analysis was subjected to corrections and calibration curves normally associated with this technique.

### 2.2. Slurry Preparation

A viscous ceramic colloidal slurry containing a high OBM solid loading (84.6 wt%) was created using a protocol adapted from prior work [18]. OBM powder was mixed with 97% pure ethylene glycol dimethacrylate (EGDMA, TCI America, Portland, OR, USA) monomer and polymeric dispersant (Solsperse AC7550, Lubrizol Advanced Materials, Inc., Cleveland, OH, USA) to enable viscous extrusion in photocasting. Diphenyl(2,4,6-trimethylbenzoyl) phosphine oxide (TPO, TCI America, Portland, OR, USA) was added to initiate photopolymerization during the fabrication process. The slurry was hand-mixed until it was homogeneous then milled in phases in a PQ-N04 planetary ball mill (Across International, Livingston, NJ, USA) using zirconia milling jars with zirconia milling media at a 1:2 powder-to-media ratio for a total of 5 hours. The slurry was then retrieved and stored in sealed containers in the dark for subsequent use. 

A similar viscous ceramic colloidal slurry containing a high solid loading (81.8 wt%) of TCP was created for the control group using a protocol adapted from prior work [18]. TCP powder (Berkeley Advanced Biomaterials, LLC, Berkeley, CA, USA) with an average nanocrystal size of 100 nm was mixed with dispersant (Solplus™ D560, Lubrizol Advanced Materials, Inc., Cleveland, OH, USA), EGDMA monomers, and TPO to create the slurry. The slurry was ball-milled in phases using zirconia milling media at a 2:5 powder-to-media ratio for a total of 10 hours and then stored in sealed containers in the dark for subsequent use.

The dynamic viscosity of the slurry was measured using an ARES G1 rheometer (TA Instruments, New Castle, DE, USA) equipped with 25 mm diameter parallel plates. A step-rate test with a 0.2 mm gap was used to measure the dynamic viscosity of the slurry at shear rates of 0.1, 1, 10, and 100 s^−1^. 

### 2.3. Scaffold Design and Fabrication

Cubic gyroid scaffolds were modeled using a gyroid infill pattern in open-source software [19] to create printable g-code based on the trigonometric approximation of gyroids using the equation gyroid(x,y,z)≡sin(x)cos(y) + sin(y)cos(z) + sin(z)cos(x) = 0, (1) where length, width, and height are represented by x, y, and z dimensions, respectively. Cubic gyroid scaffolds were designed with a desired relative porosity of 78% [47] and with a 12 mm dimension to account for the expected volumetric shrinkage of the scaffolds after sintering, which resulted in a final side length of 10 mm [19,45]. The gyroid design was exported as an STL file and sliced using Ultimaker Cura slicing software (Ultimaker B.V., Utrecht, The Netherlands, https://www.createeducation.com/software/cura/) to create g-codes for a layer-wise deposition path. The nozzle diameter dictated the road width of 0.406 mm, which was used in the design. Roads are defined as the continuous deposition of slurry through the printer nozzle at each layer of the green body scaffold. The resulting three-dimensional scaffold walls are labeled as scaffold struts. 

Gyroid scaffolds were photocast using a prior method [19] on a Hyrel Engine-SR (Hyrel 3D, Norcross, GA, USA) 3D printer. The slurry was loaded into a print head assembly EMO-XT (Hyrel 3D, Norcross, GA, USA) with a 22-gauge (0.406 mm) tip under limited ambient light exposure. The gyroid g-code was loaded and the scaffold was printed by continuous, layer-wise deposition of slurry at a print speed of 5 mm/s and an extrusion rate of 1600 and 1400 pulses per microliter (PPM) for both TCP and OBM slurries, respectively. Different pulses per microliter account for the variations in slurry viscosities. Photopolymerization of the scaffold was initiated by exposure to a light-emitting diode (LED) at a wavelength of 405 nm during the layer-wise deposition of the photocasting process. Once the scaffolds were completed, they were exposed to LED light for an additional 3 min. At this state, the scaffold is referred to as a green body. Dimensions were measured using a caliper and green body scaffolds were stored for subsequent sintering.

Green body scaffolds were sintered in a muffle furnace (Barnstead/Thermolyne 47900, Ramsey, MN, USA) to remove organic content using an adaptation of a prior method [19]. The scaffolds were placed inside the furnace at ambient temperature, which was increased at a ramp rate of 5 °C/min to 1200 °C and held constant for 3 hours before cooling naturally to room temperature. At this point, the scaffolds are referred to as as-sintered. The dimensions of the sintered scaffolds were measured in the x, y, and z faces using a caliper to record green body shrinkage to final as-sintered side length dimensions of approximately 10 mm.

### 2.4. Scaffold Structure Characterization

#### 2.4.1. Micro-Computed Tomography

The wall thickness, wall spacing, and relative porosity of OBM (*n* = 3) and TCP (*n* = 4) scaffolds were assessed through micro-computed tomography (micro-CT). Images were obtained with a Scanco 80 (Scanco Medical AG, Bruttisellen, Switzerland) instrument with a 37 µm voxel size and analyzed using Scanco Visualizer software with a script intended to analyze bone. Trabecular thickness (Tb.Th) and trabecular spacing (Tb.Sp) refer to the average scaffold wall thickness and average space between walls, respectively. The relative porosity of the scaffolds was obtained using the Equation $\varphi =1-\frac{BV}{TV}*100\%$, (2) where *φ* is the relative porosity of the scaffold, *BV* (bone volume) is the measurement of the actual volume of the scaffold material, and *TV* (total volume) is the absolute space occupied by the scaffold. In this work, relative porosity is referred to as porosity.

#### 2.4.2. Permeability

The Darcian permeability, *k*, of the gyroid scaffolds was measured using the Equation $k\;=\;\frac{v\mu L}{∆PA}$, (3) where *v* is the fluid flow velocity, *μ* is the fluid dynamic viscosity, *L* is the scaffold length, ∆*P* is the pressure drop, and *A* is the scaffold cross-sectional area. Darcian permeability can be calculated when flow regimes through porous structures have Reynolds numbers less than 1, meaning the viscous forces are larger than the product of the inertial forces to which the fluid is subjected within the porous structure [16].

The experimental apparatus was adapted from Santos et al. [16]. Darcian permeability coefficients of OBM and TCP scaffolds (*n* = 3 per group) were calculated at flow rates of 2, 3, 4, and 5 mL/min with triplicate measurements obtained at each flow rate across a differential pressure transducer (Validyne Engineering, P17-16-N-1, Canoga Park, CA, USA). The entire apparatus was purged with water after loading the scaffolds and the pressure of stagnant fluid was measured. Then, after 25 s of steady flow, pressure differential was measured and recorded for 15 s and the Darcian permeability was calculated.

#### 2.4.3. Compression Testing

Sintered gyroid scaffolds (*n* = 10 for TCP and *n* = 13 for OBM) underwent axial compression testing on a Tinius Olsen H1KS S-Series (Tinius Olsen, Horsham, PA, USA) equipped with a 1 kN load cell. The scaffold top and bottom faces (the z printing direction) were sanded lightly using 1000-grit sandpaper, applying even pressure to remove any protrusions from the bottom and top surfaces, to ensure faces were parallel and to assure even load distribution across the top and bottom scaffold faces. Samples were placed inside the load cell and secured with a 2 N force. Stress–strain curves were obtained from samples being compressed at a crosshead displacement rate of 0.1 mm/min until failure. Failure was defined as a 75% drop from the maximum load applied. Stress was calculated by dividing the applied load by the initial cross-sectional area of the scaffold perpendicular to the direction of testing, which was determined from the product of the x and y measurements of the sintered scaffolds. Cross-sectional area measurement was selected to normalize stress in gyroid scaffolds as true cross-sectional area varies as a function of scaffold height. Ultimate compressive strength is defined as the maximum stress reached in each curve. Apparent elastic modulus (slope from 0 to 0.5% strain), ultimate modulus (slope from 0 to maximum stress), and energy absorbed (area under the curve up until failure) were obtained from the analysis of the respective scaffold stress–strain curves using MATLAB^®^ R2023b.

### 2.5. Surface Analysis

Scaffold surface images were obtained with an SEM JSM-6500F (JEOL USA, Peabody, MA, USA) at a 12 kV voltage. Samples were coated with 10 nm of gold before being placed in a low vacuum within the SEM chamber. Average grain sizes and standard deviations were measured following the Heyn Lineal Intercept Procedure within the American Society for Testing and Materials (ASTM) standard E112-13 (2021) [48].

Contact Angle Goniometry (CAG) was used to evaluate the wettability of OBM and TCP. Disks (12 mm diameter, 1.8 mm height) were photocast using the respective slurries and sintering profile previously described. The captive bubble method was used to measure the advancing and receding contact angles of OBM and TCP disks on a contact angle goniometer (Model 190, Ramé-hart, Succasunna, NJ, USA) with a 0.2 mm Gilmont Buret needle attachment. Three trials of each measurement per disk (*n* = 4 per group) were averaged and then used to analyze the contact angle values. Contact angles greater than 90° or less than 90° determined if the disks were hydrophobic or hydrophilic, respectively. The collected angle measurements were converted from the liquid angle to the bubble angle using DROPImage CA software (Ramé-hart, Succasunna, NJ, USA).

### 2.6. Statistical Analysis

Evaluations of all experimental values comparing scaffold test groups were made using paired or heteroscedastic, two-tailed distribution Student T-test functions in Microsoft Excel^®^ (version 2502). Alpha levels of significance were denoted with “ns” for *p* > 0.05, * for *p* ≤ 0.05, ** for *p* ≤ 0.01, *** for *p* ≤ 0.001, and **** for *p* ≤ 0.0001. Mechanical comparisons between test groups featured a population of 10 and 13 samples for the control (TCP) and test (OBM) groups, respectively. Darcian permeability comparisons were made between the two scaffold groups with 3 unique samples per group evaluated at 4 independently tested flow rates. The Weibull failure probability, *F*(*x*), was assessed to assert the mechanical reliability of brittle ceramic scaffolds using the Weibull Cumulative Distribution Function (CDF) shown in the Equation [49] $F\left(x\right)=1-e^{-{\left(\frac{x}{\alpha }\right)}^{\beta }}$, (4) where *x* is the applied stress, *α* is a scaling parameter, and *β* is the measure of variability of the material strength, which is also known as the Weibull modulus.

## 3. Results

### 3.1. Powder Characterization and Rheological Behavior

Particle shape and size were measured from SEM images. β-Tricalcium phosphate (TCP) particles exhibited an elliptical shape with sizes ranging from 100 to 500 nm, which formed larger particle agglomerates. Deproteinized ovine bone mineral (OBM) particles exhibited a polyhedric shape with an average size of 1 μm. Multiple trace elements were observed in the OBM powder through ICP-MS analysis. Sodium, magnesium, and sulfur ions showed a higher concentration presence in addition to other lesser-presence elements like iron, strontium, barium, potassium, and zinc. As expected, calcium and phosphate ions were present at higher concentrations when compared to other trace elements shown in Table 1.

**Figure 1:**
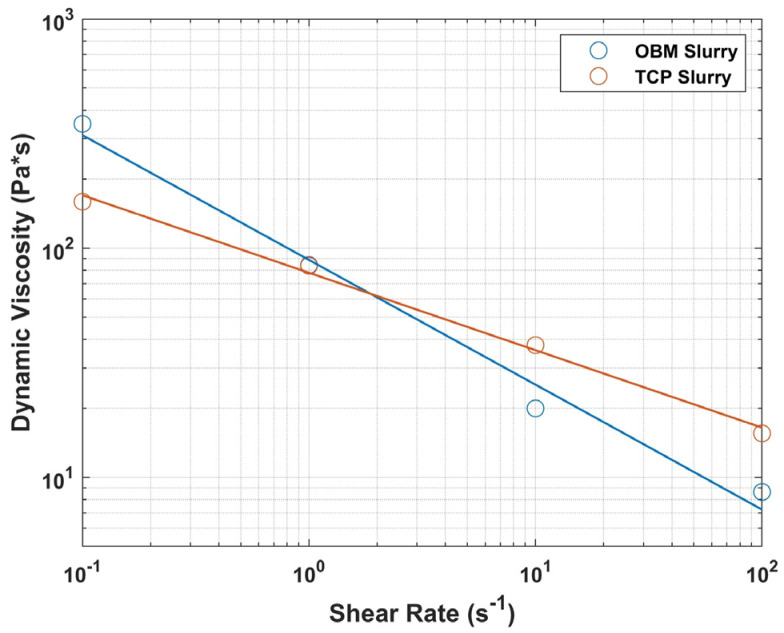
Non-Newtonian, shear-thinning behavior of slurries shown in a graph of dynamic viscosity as a logarithmic function of shear rate in addition to a linear-fit line with adjusted R-squared values of 0.9804 and 0.9926 for OBM (blue line) and TCP (red line), respectively.

The apparent viscosity of OBM and TCP slurries was obtained at different shear rates. The slurry exhibited a non-Newtonian, shear-thinning behavior in both OBM and TCP as seen in Figure 1. Such behavior is common for ceramic suspensions with high solid loading. The apparent viscosities of OBM and TCP slurries at different shear rates are shown in Table 2.

### 3.2. Scaffold Structure Characterization

#### 3.2.1. Micro-Computed Tomography

**Figure 2:**
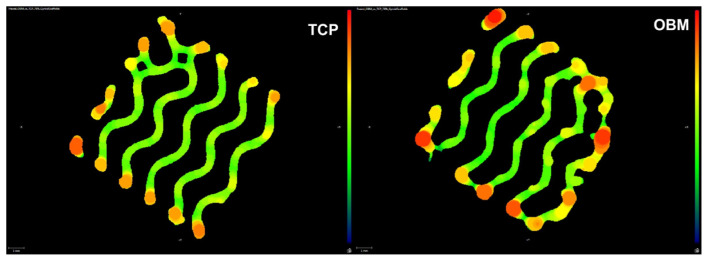
Micro-CT image slices showing wall thickness consistency as a result of the slurry photocasting process of printed OBM and TCP scaffolds.

Scaffold images were captured through micro-CT to analyze the porosity, wall thickness, and wall spacing of gyroid scaffolds dictated by viscoelastic behavior and printing rates. Figure 2 compares the uniformity between the walls of both OBM and TCP scaffolds. Wall spacing defines the average space between the scaffold walls, while wall thickness measures the average width of scaffold walls. As seen in Table 3, no significant difference was found between the average relative porosities, but a significant difference was observed between wall thickness and wall spacing. 

#### 3.2.2. Shrinkage

**Figure 3:**
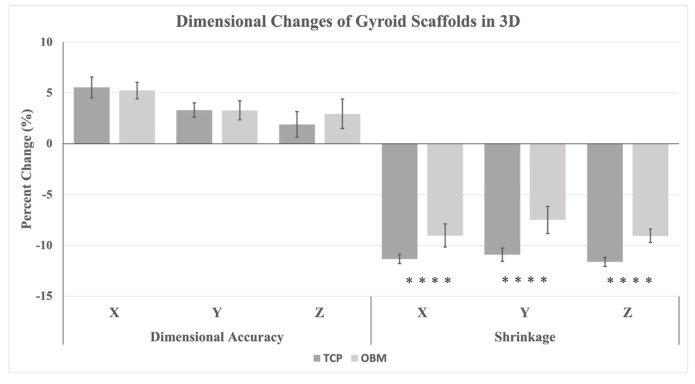
Dimensional changes of scaffolds (*n* = 14 for TCP; *n* = 16 for OBM) presented as print error and shrinkage percentages. The former represents the scaffold dimensional increase from as-designed to as-printed, while the latter represents the scaffold dimensional decrease from as-printed to as-sintered. **** for *p* ≤ 0.0001.

Dimensional changes of OBM and TCP gyroid scaffolds are summarized in Table 4 and seen in Figure 3. Green body OBM and TCP scaffolds both showed a dimensional accuracy of +5% and +3% in the *x* and *y* directions, respectively, compared to as-designed CAD dimensions of 12 mm on a side. The accuracy in the *z* direction was 3% and 2% for OBM and TCP scaffolds, respectively. No statistical significance was found between groups. Scaffolds experienced significant shrinkage in all three dimensions as a result of sintering, with ranges of 10.9–11.6% shrinkage for TCP and 7.5–9.1% for OBM, as seen in Table 4. OBM scaffolds exhibited significantly lower volumetric shrinkage of 23.5% compared to 30.2% for TCP. Such shrinkage is due to the pyrolysis of the organic components and coalescence of the hydroxyapatite ceramic components through the sintering process, which results in the densification of the final scaffold structure. 

#### 3.2.3. Permeability

**Figure 4:**
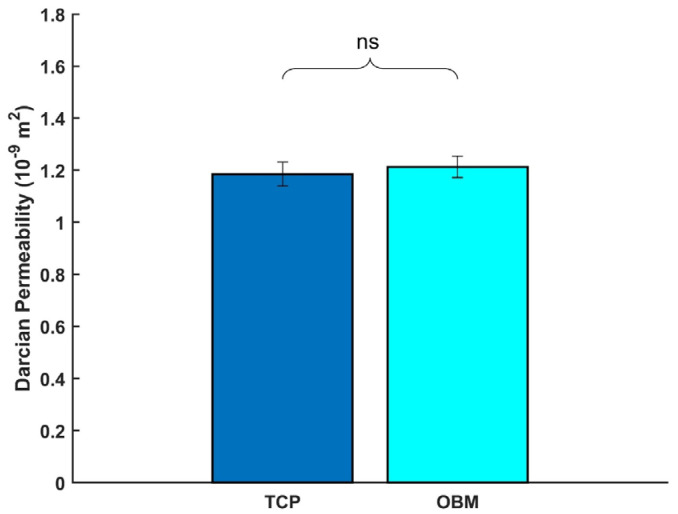
Average Darcian permeability of TCP and OBM gyroid scaffolds with standard deviation. No statistical significance (“ns”, *p* = 0.276) was found between the control (*n* = 3) and test (*n* = 3) groups.

There was no statistically significant difference between the average Darcian permeability of gyroid TCP (1.18 × 10^−9^ m^2^) and OBM (1.21 × 10^−9^ m^2^) scaffolds, as shown in Figure 4.

#### 3.2.4. Compression Testing

**Figure 5:**
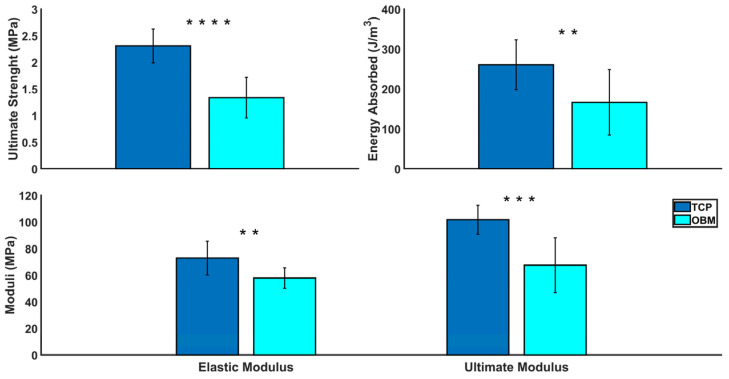
Compressive properties of TCP (*n* = 10) and OBM (*n* = 13) scaffold groups. The **top**-**left** graph shows the significant difference in ultimate strength between groups. The **top**-**right** graph shows the significant difference in the energy absorbed between groups. The **bottom** graph shows the significant differences in elastic modulus (**left**) and ultimate modulus (**right**) between groups. ** for *p* ≤ 0.01, *** for *p* ≤ 0.001, and **** for *p* ≤ 0.0001.

Compressive testing data show that TCP scaffolds exhibited significantly higher ultimate strength, elastic modulus, ultimate modulus and energy absorbed when compared to OBM scaffolds, as seen in Figure 5 and summarized in Table 5. However, both TCP and OBM scaffolds experienced similar failure strains.

### 3.3. Surface Analysis

**Figure 6:**
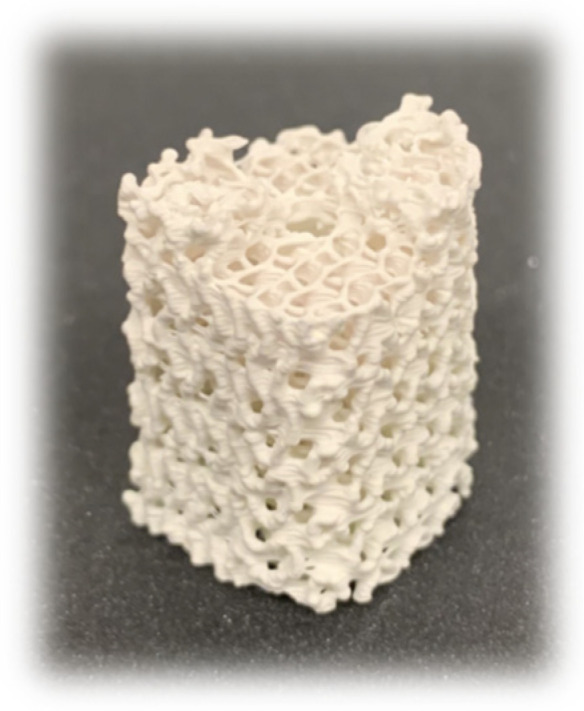
Sintered OBM gyroid scaffold half designed for patient-specific bone regeneration ovine study.

**Figure 7:**
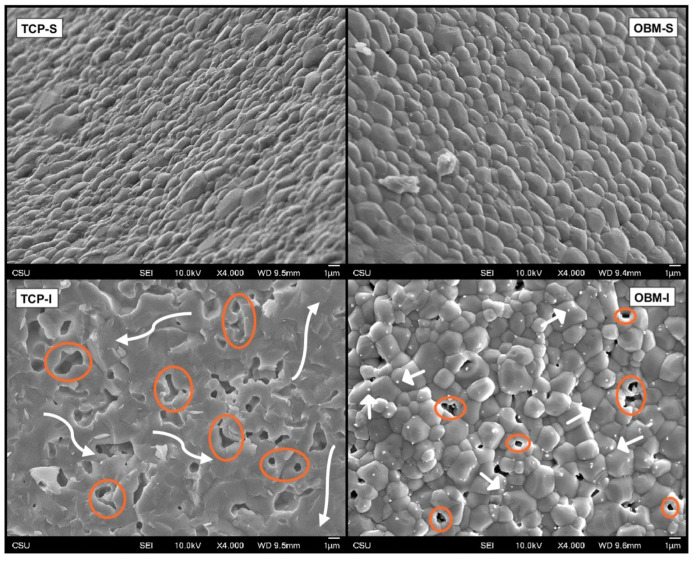
SEM images of sintered TCP (**left**) and OBM (**right**) scaffolds’ external and internal strut surfaces. The top images show scaffold external surfaces (S) with the OBM scaffold surface showing a slightly larger average grain size. Bottom images show internal strut surfaces (I) with observed micropores (highlighted ovals) of fractured scaffold where the TCP scaffold shows further grain boundary diffusion (curved arrows) when compared to the visible grain boundaries and bright nano-spots (straight arrows) of the OBM scaffold.

Figure 6 shows a sintered OBM scaffold with its corrugated surface at the macroscale. SEM images were obtained to assess the internal micro-porosity of TCP and OBM scaffolds at a microscale. The coalescence of particles reduced or eliminated micropores at the outer surface of the scaffold struts and resulted in average grain sizes of 0.88 ± 0.07 μm for TCP and 1.15 ± 0.21 μm for OBM, as seen in Figure 7. The internal coalescence of TCP scaffolds differed qualitatively from that of OBM. TCP scaffolds achieved a higher degree of coalescence, which led to less visible internal grain boundaries within the TCP struts, while the coalescence of OBM particles led to defined grain boundaries within internal surfaces. In addition, bright nano-spots are observed in the OBM internal strut surface, which are believed to be predominantly sodium and magnesium minerals [50,51]. Micropores were observed in the internal portion of both scaffolds.

**Figure 8:**
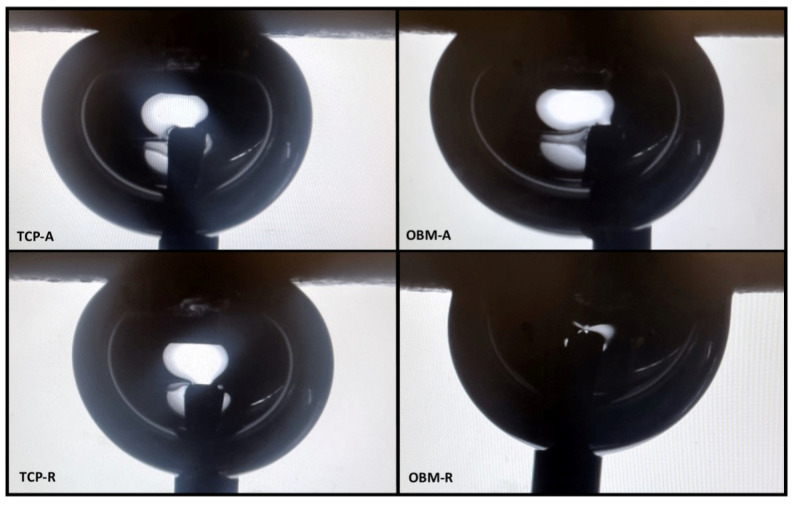
Images of advancing (A), which is measured as the surface is being wetted, and receding (R), which is measured as the surface is being de-wetted, contact angles of TCP (**left**) and OBM (**right**) photocast disks obtained through contact angle goniometry.

Scaffold surface hydrophilicity was assessed through contact angle goniometry (CAG). Contact angles are summarized in Table 6. Static, advancing, and receding contact angles were observed (Figure 8).

### 3.4. Statistical Analysis

**Figure 9:**
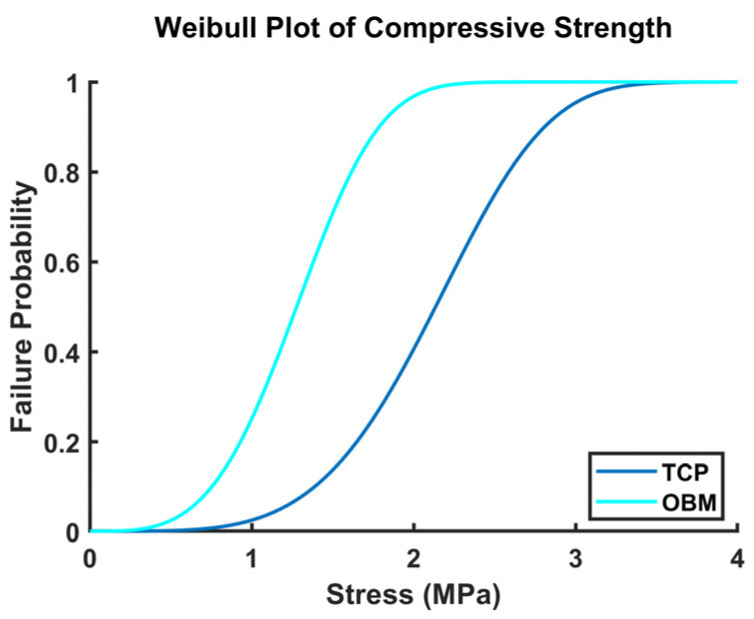
Weibull failure probability graph as a function of applied stress for both OBM and TCP scaffold groups.

Ceramic scaffold failure probability was calculated using the Weibull Cumulative Distribution Function, as seen in Figure 9. The Weibull failure probability curve as a function of applied stress indicates that OBM scaffolds have a higher chance of failing at lower applied stress values.

## 4. Discussion

TCP and OBM are fundamentally different materials in their mineral composition. Although calcium and phosphate ions are the main components of both powders, TCP is a pure synthetic biomaterial while the composition of ovine bone (OBM) is natural hydroxyapatite combined with several minerals. These minerals are found as trace elements in ovine and human long bones, including Si, S, Ti, V, K, Mn, Fe, Zn, Zr, Ag, Cd, Sn, and Sb [52]. It is known that doping synthetic TCP with minerals like Mg, Zn, Sr, and Si enhances its mechanical properties and osteogenic influence [26,27]. As a deproteinized natural bone derivative, OBM already contains the full suite of bone minerals such as Na, Mg, S, Fe, Sr, Ba, Zn, and K (Table 1). In addition, the minerals in OBM are released as cations, enabling more cell interaction sites which lead to increased osteogenesis [26,53,54]. The mineral composition of the material plays a role in determining its surface characteristics. One of the methods to evaluate the difference in material surface characteristics between TCP and OBM is to consider the wettability of the sintered surfaces. Figure 8 shows advancing and receding contact angles, which indicate respective nonpolar and polar aspects of the surface [55]. The values of static, advancing, and receding contact angles, shown in Table 6, indicate that both TCP and OBM surfaces are hydrophilic because they show contact angles that are less than 90°. However, the advancing and receding contact angles for OBM are larger, indicating OBM is more hydrophilic as the surface is wetted and de-wetted. This may be the result of the exposed cations from the minerals found on the OBM scaffold surface in addition to the hydroxyl groups in hydroxyapatite. Higher hydrophilicity tends to promote cell adhesion and proliferation on the scaffold surface [56]. Due to its innate mineral content, OBM shows promise as a suitable biomaterial candidate for bone regeneration. Hence, this work focuses on the fabrication process and characterization of OBM bone tissue engineering scaffolds.

Two factors that play important roles in 3D printing OBM scaffolds are slurry rheological behavior and apparent viscosity. These properties are directly influenced by particle characteristics such as the shape, size, size distribution, particle solid loading, and interparticle forces [57,58,59]. Particles with a more spherical shape and small size (~1 μm) with a low particle size distribution tend to have lower viscosity and enable higher solid loading. Particle shape also influences the rheological behavior of the slurry as viscosity tends to increase when particle shape deviates from sphericity [46]. Dispersant also plays an important role in rheological behavior. Figure 1 shows how both slurries exhibited shear-thinning behavior despite different dispersants being used for TCP and OBM slurries to account for differences in interparticle interaction between slurry components. Shear-thinning behavior is common for ceramic suspensions with high solid loading [59,60]. The shear-thinning behavior of the OBM slurry is attributed to the interaction of the dispersant with the micron-size OBM particles, as the dispersants enable slurry flow while preventing particle aggregation [59]. TCP slurry contained elliptical particles with a significantly lower size range (100–500 nm), which improved its flow compared to OBM’s polyhedric particle shape. These differences in rheological behavior between OBM and TCP are believed to be associated with differences in powder composition and interactions as discussed above. These factors play a crucial role in slurry formulation, which directly affects printability. Further studies in this area are recommended to better understand the intermolecular interactions between ceramic particles and organic components. 

Differences in rheological behavior between TCP- and OBM-based slurries affected the layer-wise photocasting process. Slurries had a similar apparent viscosity of 84 Pa*s at a shear rate of 1 s^−1^ but not at shear rates of 0.1, 10, and 100 s^−1^ (Table 2). These differences were accommodated by adjusting the extruded volume, which was measured in pulses per microliter (PPM) during the viscous extrusion process. OBM and TCP slurries were extruded at 1600 and 1450 PPM, respectively, to account for the different shear-thinning behavior of the two biomaterials. Higher PPM in OBM resulted in extrusion of thicker scaffold roads, which in turn produced significantly thicker walls on the sintered scaffolds, as indicated in Table 3. The width of the extruded roads varied more broadly at the scaffold edges due to the change in direction at the nozzle turning points, which led to temporary excess material extrusion referred to as “ooze”. The ooze is evident in Figure 2 where the density gradient indicates higher densities at the scaffold edges. As seen in Figure 3, both OBM and TCP green body scaffolds increased in size in all three-dimensional directions compared to the as-designed scaffold geometry, due to the ooze that formed during extrusion. The size increase varied more in the z direction than in x or y, more so in OBM scaffolds (2.95%) than in TCP scaffolds (1.9%). TCP scaffolds showed less ooze with more consistent roads in the sintered scaffold. This was probably why the TCP printing process required less user intervention than OBM and yielded green bodies with fewer print errors. On the other hand, inconsistencies in the flow behavior of OBM slurry led to less road consistency and more ooze (Figure 6), which led to more user intervention during the photocasting process. These slurry flow behavior inconsistencies and their effects during photocasting were also observed in previous work with hydroxyapatite slurries [19]. If new roads do not bond continuously to roads in a prior layer, structural weaknesses are created, which eventually become stress concentrations and failure points for the scaffold [19]. The printing errors described above decrease the final scaffold’s mechanical performance, which may explain why OBM scaffolds are weaker than TCP. Therefore, consistent, homogeneous slurry behavior is crucial to ensure reproducible, quality prints.

Green body scaffolds were sintered to create gyroid structures with internal walls, which are referred to in the micro-CT analysis. Wall thickness and spacing correlate to the scaffold porosity, which plays a crucial role in bone regeneration, facilitating mass and nutrient transport [41,61]. Sintered OBM scaffolds exhibited significantly higher average wall thickness and wall spacing (0.594 and 1.26 mm) when compared to those of sintered TCP scaffolds (0.519 and 1.18 mm). Despite differences between wall thickness and spacing, no significant difference was found between relative scaffold porosities, as seen in Table 3. The outer dimensions of OBM scaffolds were slightly larger than TCP, which would explain the similar relative porosities given the significant difference in wall thickness and spacing. The similar porosity between OBM and TCP scaffolds correlates to scaffold permeability, as it largely depends on scaffold topology and porosity. Figure 4 shows no significant difference in the permeability of TCP (1.18 × 10^−9^ m^2^) and OBM (1.21 × 10^−9^ m^2^) with a similar gyroid topology. Surface characteristics like hydrophilicity also play a role in permeability. In this study, the higher hydrophilicity of OBM discussed earlier did not seem to be sufficient to cause a measurable increase in permeability.

Prior work has shown that sintering green body scaffolds causes shrinkage due to the coalescence of the ceramic particles and the pyrolysis of the organic components [19,62,63]. Significant shrinkage occurred in both TCP and OBM scaffolds (Table 4), with TCP shrinking approximately 2% more than OBM in all three dimensions. The following factors are considered to explain the difference in shrinkage percentages: (1) The sintering profile used for both test and control groups may not have allowed for the proper pyrolysis of the organic components and calcination of the printed scaffolds. Proper calcination of scaffolds could be achieved through further particle coalescence and scaffold densification [64]. (2) Air pockets can be created by not allowing enough time for the volatile organic components to escape the scaffold at their evaporating temperature [65,66]. These could lead to eventual crack defects within the scaffold. (3) The effect and importance of the powder particle sizes are evident in the subsequent grain sizes achieved post-sintering. The average particle sizes of TCP (100–500 nm) and OBM (1 μm) correlated to the mean grain sizes of TCP (0.88 ± 0.07 μm) and OBM (1.15 ± 0.21 μm) sintered scaffold surfaces, which were significantly different (*p* = 0.002). Figure 7 shows the formation of grain boundaries on the internal OBM scaffold surface, in addition to bright nano-spots which are believed to be sodium and magnesium mineral components, as seen in previous work [50,51], while further qualitative grain boundary diffusion is observed on the internal TCP scaffold surface [67]. Such additional densification change can be attributed to the TCP phase transition from β to α, which starts at 1200 °C [68]. A combination of these factors could explain the difference in shrinkage of OBM and TCP.

The photocasting process and sintering profile affect the mechanical properties of sintered scaffolds. In addition, proper bonding between layers during photocasting is necessary to maintain overall structural integrity. Figure 5 shows that TCP gyroid scaffolds exhibited significantly higher mechanical properties than OBM. These results are summarized in Table 5. TCP scaffolds absorbed more energy and exhibited significantly higher compressive strength compared to their OBM counterparts. This finding is consistent with prior observation that porous scaffolds usually exhibit a direct linear relationship between scaffold energy absorption and mechanical loading [69]. The compressive strengths of both TCP and OBM scaffolds (2.31 ± 0.32 and 1.34 ± 0.39 MPa, respectively) fell in the lower end of the range of compressive strength of trabecular human bone (1.4–6.8 MPa) found in the literature [70]. Previous studies on human trabecular bone show that samples with increasing bone volume fractions result in higher apparent elastic modulus and sample strength [71], which is due in part to the natural hierarchical structure and variable density of natural bone [72]. Gyroid porous structures were chosen for this study due to their close resemblance to trabecular bone, which includes relatively high mechanical energy absorption [14], high surface area per volume, and a continuous, interconnected internal porous structure [10,11,13]. In addition, TCP scaffolds had significantly higher elastic modulus and ultimate modulus which were obtained from the stress–strain curves. All OBM stress–strain curves showed a self-reinforcing behavior, while only half of the TCP curves exhibited this behavior. TCP curves resembled more monatomic brittle behavior while OBM was more quasi-brittle. The variance in the photocasting process discussed earlier could be one of the reasons why all stress–strain curves for OBM scaffolds exhibited such behavior, which is similar to the phenomenon discussed in prior work with hydroxyapatite gyroid scaffolds [19,73]. The TCP scaffold’s monatomic brittle behavior could be associated with the lower average grain size and further internal densification of TCP particles when compared to OBM particles and their densification. Smaller grain sizes act as effective dislocation and crack propagation interruption, leading to shorter cracks and fewer failure points within the scaffolds [49,74]. Hence, fewer dislocations and interruptions of crack propagations hypothetically lead to relatively stronger TCP scaffolds. The Weibull failure probability graph (Figure 9) showed that OBM scaffolds were more likely to fail at lower stress values than TCP, even though both OBM and TCP had similar failure strain values, confirming the relatively lower mechanical reliability of OBM scaffolds. We hypothesize that the higher mechanical properties of TCP scaffolds result from differences in fabrication processes in addition to differences in particle characteristics and mineral composition between pure, synthetic TCP and natural bone mineral.

## 5. Conclusions

To the best of our knowledge, this paper presents the first 3D printing of porous scaffolds from naturally derived bone mineral. OBM powder composition and particle characteristics were evaluated. Powder composition had a substantial effect on the rheological behavior of the OBM slurry and subsequently on printability because of the complex, natural mineral composition of OBM compared to the synthetic nature of TCP. Rheological behavior dictated the scaffold printing process and print accuracy, which influenced the post-sintered scaffold mechanical properties. Mechanical properties of sintered TCP and OBM gyroid scaffolds were assessed and compared. TCP was stronger than OBM in all comparisons except failure strain. Furthermore, OBM scaffolds were more likely to fail at lower stress values when compared to TCP. These results are attributed to differences in innate powder composition, slurry behavior, user intervention during fabrication, and sintering profile. Better understanding of rheological interactions and optimization of the scaffold sintering profile are recommended to improve printing reliability and to enhance mechanical properties. The compressive properties of both TCP and OBM scaffolds are at the lower end of the range of trabecular human bone, suggesting that porous gyroid OBM scaffolds may be suitable for bone regeneration applications. Future work should include in vitro cell studies and in vivo implantation to evaluate biomedical suitability.

## Figures and Tables

**Table 1:** Concentrations of trace elements in OBM powder measured with ICP-MS.

Element	Concentration (µg/g)
Ca	106.046
P	58.929
Na	2.299
Mg	2.243
S	0.893
Fe	0.270
Sr	0.228
Ba	0.185
Zn	0.036
K	0.018

**Table 2:** Summary of OBM and TCP slurry apparent viscosities at different shear rates through parallel plate rheometry.

Shear Rate (s^−1^)	TCP Slurry (Pa*s)	OBM Slurry (Pa*s)
0.1	159.5	348.7
1	83.8	84.3
10	37.7	20.0
100	15.5	8.64

**Table 3:** Summary of wall thickness, wall spacing, and porosity of sintered OBM and TCP scaffolds from micro-CT images. ns for *p* > 0.05 and ** for *p* ≤ 0.01.

Measurement	TCP (*n* = 4) Mean ± SD	OBM (*n* = 3) Mean ± SD	Significance
Relative Porosity (%)	66.37 ± 1.19	67.12 ± 0.77	ns
Average Wall Thickness (mm)	0.52 ± 0.17	0.59 ± 0.16	**
Average Wall Spacing (mm)	1.18 ± 0.21	1.26 ± 0.24	**

**Table 4:** Summary of print dimensional accuracy and shrinkage percentages of OBM and TCP green body and sintered scaffolds. ns for *p* > 0.05 and **** for *p* ≤ 0.0001.

		TCP (*n* = 14) Mean ± SD	OBM (*n* = 16) Mean ± SD	Significance
	X	5.55 ± 1.04	5.24 ± 0.82	ns
Dimensional Accuracy (%)	Y	3.32 ± 0.69	3.29 ± 0.93	ns
	Z	1.90 ± 1.27	2.95 ± 1.45	ns
	X	−11.33 ± 0.45	−9.02 ± 1.13	****
Shrinkage (%)	Y	−10.90 ± 0.65	−7.48 ± 1.33	****
	Z	−11.62 ± 0.45	−9.05 ± 0.65	****

**Table 5:** Summary of compressive properties of TCP and OBM gyroid scaffolds. ns for *p* > 0.05, ** for *p* ≤ 0.01, *** for *p* ≤ 0.001, and **** for *p* ≤ 0.0001.

Measurements	TCP (*n* = 10) Mean ± SD	OBM (*n* = 13) Mean ± SD	Significance
Compressive Strength (MPa)	2.31 ± 0.32	1.34 ± 0.39	****
Elastic Modulus (MPa)	72.9 ± 12.8	57.9 ± 7.76	**
Ultimate Modulus (MPa)	101.7 ± 10.9	67.5 ± 20.5	***
Energy Absorbed (J/m^3^)	260.9 ± 62.5	166.6 ± 82.2	**
Failure Strain (%)	2.38 ± 0.40	2.27 ± 0.86	ns

**Table 6:** Summary of contact angle measurements of TCP and OBM disks with no significant difference between groups.

Contact Angle Measurement	TCP (*n* = 4) Mean ± SD	OBM (*n* = 4) Mean ± SD
Static	42.5° ± 4.4°	41.1° ± 3.6°
Advancing	35.5° ± 4.4°	40.1° ± 5.1°
Receding	66.8° ± 9.0°	74.2° ± 5.5°

## Data Availability

The original contributions presented in the study are included in the article; further inquiries can be directed to the corresponding author.
